# A 'meta-analysis' of effects of post-hatch food and water deprivation on development, performance and welfare of chickens

**DOI:** 10.1371/journal.pone.0189350

**Published:** 2017-12-13

**Authors:** Ingrid C. de Jong, Johan van Riel, Marc B. M. Bracke, Henry van den Brand

**Affiliations:** 1 Wageningen Livestock Research, Wageningen University and Research, Wageningen, The Netherlands; 2 Adaptation Physiology Group,Wageningen University and Research, Wageningen, The Netherlands; Leibniz-Institut fur Pflanzengenetik und Kulturpflanzenforschung Gatersleben, GERMANY

## Abstract

A ‘meta-analysis’ was performed to determine effects of post-hatch food and water deprivation (PHFWD) on chicken development, performance and welfare (including health). Two types of meta-analysis were performed on peer-reviewed scientific publications: a quantitative ‘meta-analysis’ (MA) and a qualitative analysis (QA). Previously reported effects of PHFWD were quantified in the MA, for variables related to performance, mortality and relative yolk sac weight. The QA counted the number of studies reporting (non-)significant effects when five or more records were available in the data set (i.e. relative heart, liver and pancreas weight; plasma T3, T4 and glucose concentrations; relative duodenum, jejunum and ileum weight; duodenum, jejunum and ileum length; and villus height and crypt depth in duodenum, jejunum and ileum). MA results indicated that 24 hours of PHFWD (i.e. ≥12–36 hours) or more resulted in significantly lower body weights compared to early-fed chickens up to six weeks of age. Body weights and food intake were more reduced as durations of PHFWD (24, 48, 72, ≥84 hours) increased. Feed conversion rate increased in chickens up to 21 and 42 days of age after ≥84 hours PHFWD in comparison with chickens fed earlier. Total mortality at day 42 was higher in chickens after 48 hours PHFWD compared to early fed chickens or chickens after 24 hours PHFWD. First week mortality was higher in chickens after ≥84 hours PHFWD than in early fed chickens. The MA for relative yolk sac weight was inconclusive for PHFWD. The QA for plasma T3, T4 and glucose concentrations indicated mainly short-term decreases in T3 and glucose in PHFWD chickens compared to early fed chickens, and no effects of PHFWD on T4 concentrations. Relative weights of liver, pancreas and heart were lower after PHFWD, but only in the first week of life. A retarded development of gut segments (duodenum, jejunum and ileum) was found in the first week of life, measured as shorter, lower relative weight, and lower villus height and crypt depth. It is concluded that 48 hours (≥36–60 hours) PHFWD leads to lower body weights and higher total mortality in chickens up to six weeks of age, the latter suggesting compromised chicken welfare, but effects of PHFWD on organ development and physiological status appear to be mainly short-term.

## Introduction

In a commercial hatchery, chickens and poults (referred to as ‘chickens’) usually hatch over a period of 24–48 hours (the ‘hatch window’) [1–3]. Newly-hatched chickens remain in the incubator until almost all have hatched, after which all chickens are collected. This is usually performed at day 21.5 of incubation (broilers and laying hens). After collection, also termed ‘pulling’, the chickens undergo hatchery treatments, such as selection of second-grade chickens, vaccination, sex determination and/or sorting. Thereafter, chickens are transported to the farm. From the moment of hatching until placement at the farm, food and water is usually withheld. The duration of this period depends on the hatch window, hatchery treatments and transport duration [2–5]. The time until first food and water intake (‘holding period’) may take up to 72 hours where long transportion distances are involved [2, 3].

Chickens have been considered to be able to survive on the yolk sac reserves for a period of up to approximately 72 hours after hatching [6]. However, while the yolk sac reserves may be sufficient for survival, it can be disputed whether or not all requirements have been met during the first 72 hours post-hatching. Willemsen et al. [3] suggested that post-hatch food and water deprivation (PHFWD) may have long term negative consequences for chicken welfare, and that their behavioural and physiological requirement for food and water may not be met under current commercial conditions [3].

In recent decades, studies have examined the effects of PHFWD on chicken performance, development and welfare (see e.g., [3, 4, 7]). However, these studies have provided ambigious results and a more quantitative (meta-) analysis of existing data is lacking. This study aimed to assess the effects of PHFWD on poultry performance and welfare. More specifically the objective was to identify and quantify the measurable impacts of PHFWD on the development, performance and welfare of (especially broiler) chickens based on (the statistical analyses of reported experiments in) the existing scientific literature.

## Materials and methods

### Literature search

Peer reviewed articles, published between 2000 and 2016 were included in a quantitative ‘meta-analysis’ (MA) and in a qualitative analysis (QA). Between October 5–28, 2016 literature searches were done using Web of Science. The following key words were used: (broiler* OR laying hen OR turkey) AND (early food* OR early fasting OR post hatch food*) AND >2000 publication year. In addition, [8] was consulted for additional articles and cross-references were used. Studies on broiler chickens, laying hen chickens and turkey poults were included.

### Selection of papers

Studies in which access to food and water was confounded with another factor (e.g., housing system or food supplement) were not included in the analyses. Papers from which it was impossible to extract reliable quantitative scores (e.g., when only graphs were provided) and papers related to in-ovo feeding were also excluded.

For each selected paper, moment of first food and water supply (category 1 to 3, see section ‘Definitions’) was identified. Additionally, effects of PHFWD on the different variables determined in the study were registered in terms of significance (P<0.05, yes/no) and whether they were higher or lower in the PHFWD chickens. When more treatments were included in a paper, they were all taken into account, for example when different durations of PHFWD were applied.

Papers used for the MA were distinguished into two categories; 1) papers in which chickens were deprived of both food and water after hatching (FW) and papers in which chickens were deprived of food only (FO) (while water was provided) after hatching. Papers in which only water was deprived after hatching (but not food), were not included in the MA, because the number of papers was insufficient.

MA was performed if sufficient records (N≥10) were available for a specific variable. Less than 10 records may result in unreliable effects, because in that case a single study can have a dominant effect on the results of the MA. In the MA, a regression analysis was used (see below) to combine the results from multiple studies to increase power and to provide estimates of the size of effects [9].

The duration of PHFWD varied among papers. Because of that in both the MA and QA, categories of PHFWD duration were made; 0 to 12, ≥12 to 36, ≥36 to 60, ≥60 to 84 and ≥84 hours. These categories are reported as 0, 24, 48, 72 and ≥84 hours. Thus, studies with 12 hours deprivation were only used in the category ≥12 to 36 hours, and likewise for the other categories.

Each record in the spreadsheet represented the result of a specific combination of treatments within an experiment. In case the experiment had a non-factorial design this implied that treatment average values for the specified variable (e.g., body weight, mortality, etc.) were collected in the spreadsheet. In case of a factorial design, data were collected in the spreadsheet only if effects were reported on interaction level. Thus, one paper could report more than one experiment, and also (in case of a significant interaction within an experiment) more than one record with the same PHFWD duration within an experiment. Each relevant experiment had at least two treatments of which one treatment was 0 hours PHFWD. This generated multiple records per scientific paper, depending on the number of treatments and experiments involved.

Recorded data were also classified into production variables such as body weight (gain), yolk-free body mass, food intake, feed conversion ratio (FCR) and mortality, and variables related to health (e.g., morbidity and immunological parameters), behaviour, physiology (e.g., hormones and organ weights), and intestinal development (e.g., villus height, crypt depth, relative length and microbiota composition).

### Definitions

In this section we define chicken age, and we define the various concepts integral to (the objective of) this paper, namely MA, PHFWD, welfare, performance and development.

In the selected papers, the moment of first food and water supply after hatching differed and was not always indicated clearly. Therefore, papers were classified into three categories of chicken age:

Category 1: The biological age [5] of the chickens was known; chickens were collected periodically (e.g., every 2 to 6 hours) from the incubator during the hatching process. When it was indicated that the down was still wet at collection, this was classified as collection within 3 hours after hatching.

Category 2: The chronological age [5] of the chickens was known. Chickens were collected at pulling. This included the term ‘day-old chickens’.

Category 3: The age of the chickens was unknown. For these experiments, it was not clear at which age chickens were being fed (e.g., when the term ‘newly hatched chickens’ was used).

In this paper we refer to our analysis as a ‘meta-analysis’, because we did not do our analysis on the (aggregate of) primary datasets of the underlying studies. Instead, we used the outcomes of the statistical analyses (average values) reported in the scientific papers as the basis for our ‘meta-analysis’ (MA).

The abbreviation ‘PHFWD’, which stands for “post-hatch food water deprivation” more specifically refers to “post-hatch FW or FO deprivation”, i.e. post-hatch food and water deprivation (FW) or food only (but not water) deprivation (FO). This is because the treatment involving deprivation of water only was not reported sufficiently frequent in the scientific literature.

Finally, we felt a need to specify several concepts related to the objective of this paper (animal welfare, performance and development). We define animal welfare as the quality of life as perceived by the animals themselves [10]. The word ‘performance’ here refers to production performance, which for broilers and poults, refers to aspects related to growth and variables like FCR, etc. Development refers to the birds’ transition over time. In this paper the birds’ development is primarily related to (various aspects of bird) physiology.

### ‘Meta-analysis’ (MA)

MA was performed for the effects of PHFWD duration on body weight, cumulative food intake, FCR and mortality at day 7 (±1), day 21 (±1) and day 42 (±2) of age. These three ages were most commonly used in studies on PHFWD. Log-transformation of these dependent variables was needed to account for higher variability of average treatment values in case of a higher level in a particular experiment, and an assumption of comparable relative treatment effects was made. Due to lacking information of standard errors in many papers, a inverse-variance weighting analysis was not conducted. Inclusion of only papers with information on standard errors would have resulted in too few papers to perform the MA.

Three analyses were performed: for all categories of papers (category 1+2+3), for category 1+2 only (biological and chronological age of the chickens known) and for category 1 only (biological age of chickens known).

For relative yolk sac weight, the analysis was performed for day 1 to 6 of age, in which all categories of papers (category 1+2+3) were included, because there were insufficient records to separately analyse category 1 or category 1+2 papers. We performed a regression analysis in Genstat [11], using a REML procedure, separately for each PHFWD category (1 to 3). Pairwise comparisons were performed with Fischer’s LSD test.

The final model used for estimation of main effects of PHFWD duration was:

$${\underline{Y}}_{ijkl}=\mu +\alpha_{l}+{\underline{\varepsilon }}_{i}+{\underline{\varepsilon }}_{ij}+{\underline{\varepsilon }}_{ijk}+{\underline{\varepsilon }}_{ijkl}$$ [Model 1]

Where:

${\underline{Y}}_{ijkl}$        : LOG-transformed dependent variable (body weight, cumulative food intake, FCR, mortality or relative yolk sac weight) from paper i, experiment j (within paper), factorial treatments kj (within experiment) and level of duration of food deprivation l (within experiment).
*μ*          : mean value for reference (PHFWD = 0 hours)
*α*_*l*_          : effect of duration of food deprivation compared to µ; i = 24 hours, 48 hours, 72 hours, ≥84 hours
${\underline{\varepsilon }}_{i},{\underline{\varepsilon }}_{ij}$        : random effects of paper *i*, resp. experiment *j (within i)*.
${\underline{\varepsilon }}_{ijk}$        : random effects of factorial treatment *k* (in case of factorial design) within experiment *j* of paper *i*.
${\underline{\varepsilon }}_{ijkl}$        : random residual variance.

Relative values of different durations of food deprivation, compared to the situation with PHFWD = 0 hours, were derived from the average values of each level of PHFWD, where the reference level (PHFWD = 0 hours) is 100 (percent): RE = 100 * $e^{\alpha_{l}}$.

For the parameters with sufficient records Model 1 was extended with the factor FW (food and water deprivation) or FO (food only deprivation), as well as with the interaction between FW/FO on the one hand and the duration of PHFWD on the other hand (Model 2). Preliminary analyses demonstrated that an interaction existed between FW/FO and duration of PHFWD only for relative residual yolk weight. For none of the other variables this interaction was found nor a difference between FW and FO. Consequently, effects of FW and FO studies were combined in the results, except for relative residual yolk weight.

$${\underline{Y}}_{ijkl}=\mu +\alpha_{l}+\beta_{m}+{\left(\alpha \beta \right)}_{lm}+{\underline{\varepsilon }}_{i}+{\underline{\varepsilon }}_{ij}+{\underline{\varepsilon }}_{ijk}+{\underline{\varepsilon }}_{ijkl}$$ [Model 2]

Where:

*β*_*m*_,*αβ*_*lm*_        : resp. effect of FW/FO and interaction effect between FW/FO and PHFWD

### Qualitative analysis (QA)

In the QA, the number of significantly higher, significantly lower and non-significant records were counted for all variables of which five records or more, but less than 10 records, were present for one or more ages. In the QA, contrasts between 0–24 hours PHFWD, 0–48 hours PHFWD and 0–72 hours PHFWD were investigated. Similar classes were used as in the MA to overcome the wide variation in durations of PHFWD reported in the literature, i.e. 0–12 hours, ≥12–36 hours, ≥36–60 hours, ≥60–84 hours, these were likewise labelled as 0, 24, 48 and 72 hours. QA included all three categories of papers (biological age, chronological age, age not defined) and effects of PHFWD are presented for day 7 (±1), day 14 (±1), day 21(±1), day 28 (±1), day 35 (±1) and day 42 (±2). For relative weights of heart, liver, pancreas, duodenum, jejunum and ileum, relative lengths of duodenum, jejunum, ileum, villi height and crypt depth in duodenum, jejunum and ileum and plasma glucose concentrations, results are described, and graphically presented in supporting information S1–S6 Figs for days 1 to 6 of age and for 1 to 6 weeks of age. For plasma T3 and T4 concentrations results are only described, and graphically presented for T3 in supporting information S6 Fig for days 1 to 6 of age. No further statistical analysis was done in the QA, only counts of records are presented in S1–S6 Figs.

Two tables were generated (not shown). First, a discrimination was made between FW (food and water deprived/provided at the same time) and FO (deprivation of food only; water available immediately after hatching regardless of the time of feeding) studies. Secondly, no discrimination was made between FW and FO studies. As a separation of FW and FO studies did generally not lead to different results, only the results of QA of all records (including both FW and FO studies) are shown in S1–S6 Figs.

## Results

### Literature screening

**Fig 1:**
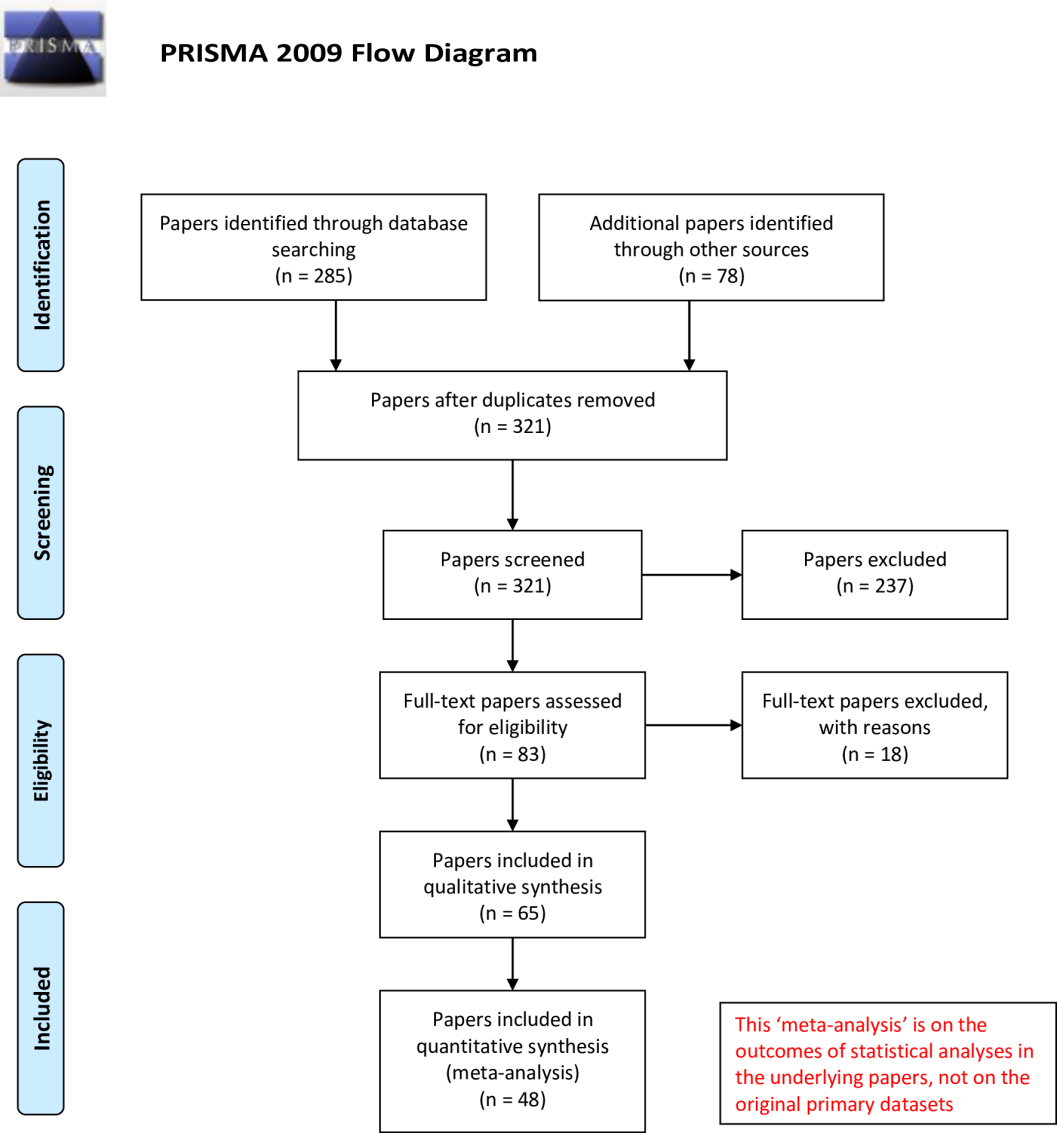
Prisma flow diagram. This diagram presents the number of scientific papers identified, screened and selected for MA and QA.

The literature searches resulted in a total of 83 scientific papers reporting experiments on PHFWD potentially suitable for a ‘meta-analysis’. After screening, 65 papers were included in the QA and 48 in the MA according to the criteria as described above (Fig 1). Each scientific paper could generate multiple records in the database, depending on the number of treatments and experiments involved, as explained above.

MA included the following studies on body weight: [12–54]; food intake: [13, 21, 22, 36–38, 40, 43, 44, 48–52, 55]; FCR: [12, 13, 20, 21, 36, 38–40, 43, 47–57]; mortality: [15, 17, 39, 40, 47–51, 54, 57, 58]; and relative yolk sac weight: [34, 37, 51, 59].

The 65 studies included in the QA (using the requirement that at least five records had to be available for a single variable to be included in the QA) resulted in QA being based on the following publications: [1, 12–75]. As the QA of production variables and mortality did not generate new information compared to the MA, results of the QA for these parameters are not shown.

### ‘Meta-analysis’ (MA)

Table 1 shows the results of the MA for the combined categories of scientific papers (category 1+2+3) for the combined FW and FO treatments (since neither of these was not found to interact with PHFWD duration; see above). MA of all categories of papers showed similar results compared to MA of only category 1 and category 1+2 papers, and results of MA for these categories of papers are therefore not presented.

**Table 1:** Effects of various durations of post-hatch food (deprivation or food and) water deprivation (PHFWD) (0, 24, 48, 72 and ≥84 hours) on body weight (BW), feed conversion ratio (FCR), cumulative food intake (FI) and total mortality at day 7, 21 and 42 of age. Average values for the various variables are expressed relative to 0 hours PHFWD, which is set at 100%.

	Relative value after food and water deprivation for^1^						
Treatment	0 hours (0–12 hours)	24 hours (≥12–36 hours)	48 hours (≥36–60 hours)	72 hours (≥60–84 hours)	≥84 hours	P value^2^	N^3^
**BW day 7**	100^a^	92.8^b^	83.0^c^	73.1^d^	51.6^e^	<0.001	204
**BW day 21**	100^a^	95.0^b^	89.3^c^	79.5^d^	*^4^	<0.001	82
**BW day 42**	100^a^	97.4^b^	94.5^c^	91.7^c^	*	<0.001	50
**FCR day 0–7**	100	99.3	103.5	*	*	NS	37
**FCR day 0–21**	100^b^	99.6^b^	98.7^b^	106.1^ab^	110.4^a^	0.013	57
**FCR day 0–42**	100^b^	99.9^b^	100.1^b^	103.8^b^	110.3^a^	<0.001	47
**FI day 0–7**	100^a^	92.1^a^	67.4^b^	63.5^b^	*	<0.001	37
**FI day 0–21**	100^a^	95.4^a^	87.3^b^	78.4^b^	*	<0.001	39
**FI day 0–42**	100^a^	98.0^a^	95.1^b^	89.2^b^	*	<0.001	33
**Mortality day 0–7**	100^bc^	81.4^c^	143^bc^	226^b^	827^a^	<0.001	39
**Mortality day 0–21**	100	102.3	200	*	*	NS	6
**Mortality day 0–42**	100^b^	100.3^b^	156^a^	*	*	0.003	29

^1^ Values within a row lacking a common superscript differ significantly (P<0.05).

^2^ P-value for effect of PHFWD.

^3^ N: number of records in the database.

^4^ A * in a cell indicates that insufficient records were available to do the analysis.

Prolonged duration of PHFWD resulted in lower body weight at day 7, 21 and 42 (all P<0.001) compared to early fed chickens. Food intake from day 0 to 7, day 0 to 21 and day 0 to 42 was lower after 48 or more hours of PHFWD (all P<0.001) compared to early fed chickens (0 hours of PHFWD). FCR between day 0 and 7 was not affected by PHFWD, wheras it was higher between day 0 and 21 (P = 0.013) and between day 0 and 42 (P<0.001) after ≥84 hours of PHFWD compared to 0 hours of PHFWD. Mortality between day 0 and 7 was higher after ≥ 84 hours of PHFWD compared to 0 hours of PHFWD (P<0.001). Mortality between day 0 and 21 was not affected by duration of PHFWD and mortality between day 0 and 42 was higher after 48 hours of PHFWD compared to 0 hours of PHFWD (P = 0.003).

The MA for relative yolk sac weight included all categories of scientific studies (category 1+2+3) (Table 2). A significant interaction was found between food and water deprivation type (FW/FO) and duration of PHFWD for relative yolk sac weight at three days of age (P<0.001). Chickens deprived of both food and water (FW) for 72 hours had a lower relative yolk sac weight than 0 hours PHFWD chickens, whereas in chickens deprived of only food (FO) the opposite was found.

**Table 2:** Effects of various durations of post-hatch food (deprivation or food and) water deprivation (PHFWD) (0, 24, 48, 72 hours) on relative yolk sac weight at 3 days of age for chickens deprived of both food and water (FW) and chickens deprived of only food (FO). Average values of relative yolk sac weight are expressed as relative values compared to 0 hours (no food and water deprivation), which is set at 100. N = 19 records.

	Relative value after post-hatch food and water deprivation for a period of^1^			
Treatment	0 hours (0–12 hours)	24 hours (≥12–36 hours)	48 hours (≥36–60 hours)	72 hours (≥60–84 hours)
**Food and water deprivation (FW)**	100^a^	118.0^a^	121.8^a^	84.6^a^
**Food deprivation only (FO)**	100^a^	*^2^	*^2^	219^b^

^1^ Values lacking a common superscript differ significantly (P<0.05).

^2^ A * in a cell indicates that insufficient records were available to do the analysis.

### Qualitative analysis

#### Relative organ weights

Most records demonstrated a lack of effect of PHFWD on relative liver, pancreas and heart weight, with a few records demonstrating a positive or negative effect (S1 Fig). Negative effects have mainly been found in the first week of age. It should be noted that the number of records was limited per duration of PHFWD and age.

#### Intestinal development

Most records in the QA demonstrated no effect of PHFWD on intestinal development expressed as duodenal, jejunal and ileal length and relative weight (S2 Fig (length) and S3 Fig (weight)). Within the first week of age, PHFWD only showed negative effects or no effects on intestinal development, with only one record demonstrating a positive exception for ileal length and relative duodenal weight after 48 hours of PHFWD at 5 days of age. For ileal length, some positive records were found of PHFWD, whereas for relative jejunal weight and relative ileal weight some positive records and some negative records were found of PHFWD. However, the number of records was very limited for most durations of PHFWD and ages (≤ 6 records per duration per age).

The QA for duodenum, jejunum and ileum villi heights and crypt depths demonstrated comparable results as found for the relative intestinal weights and lengths (S4 Fig (villi heights) and S5 Fig (crypt depths)). Most records did not demonstrate a significant effect or demonstrated a negative effect of PHFWD on villi heights and crypt depths, but also a few records demonstrated positive effects. Effects were particularly found in the first week of age (S4 Fig and S5 Fig). However, the number of records per duration of PHFWD and age was limited (≤ 6 per duration and age).

#### Plasma T3, T4 and glucose concentration

Only a few studies investigated effects of PHFWD on plasma glucose, T3 and T4 concentration levels. On the first days of age some negative effects (numerically lower values) of PHFWD were found for glucse and T3 levels, but at later ages, effects were lacking or even higher values were found (S6 Fig). However, again the number of records was very limited. No effects of PHFWD were found on T4 levels in the first week of age (data not shown because of an insufficient number of records (N = 4)).

## Discussion

### Selection of studies and variables

A wide variety of variables related to PHFWD are described in the scientific literature. However, there was only sufficient data available on body weight, food intake, FCR, mortality and relative yolk sac weight to perform the MA. Other variables were included in the QA when at least five records were available. The less frequently studied variables relevant for poultry welfare assessment are discussed here (for a more extensive discussion see [76]).

In most studies, chickens were deprived of both food and water (FW) after hatching; few papers [1, 20] subjected chickens to various durations of water deprivation without food [20, 34, 77], or sometimes papers included a treatment group with immediate access to water after hatching (FO) [1, 12, 14, 20, 25, 26, 34, 39, 53, 57, 59, 69]. This allowed us in the MA to discriminate between treatments that deprived chickens of food only (FO), by providing all chickens with water immediately after hatching, and treatments depriving chickens of both food and water (FW) by providing food and water simultaneously following the deprivation period. In the QA, very few records were available for FO. Therefore, and because the MA did not show major effects of FO, we decided to combine the results of FO and FW treatment groups.

The QA only summarises findings from several studies, but does not combine these data for a more powerful/quantitative analysis. The QA was performed when between at least five to 10 records per variable were available, but for most of the specific treatment by age combinations less than five records were available. Therefore, caution is advised when considering the results from these QA’s.

### Effect of PHFWD on poultry welfare

In the current study, effects of post-hatch food (deprivation or food) and water deprivation (PHFWD) on poultry welfare related variables (including performance, development and mortality) were analysed, using a quantitative ‘meta-analysis’ (MA) and a more qualitative analysis (QA). The objective being to determine whether or not performance, or also poultry welfare can be affected by PHFWD. To assess welfare, i.e. the animals’ quality of life, animal scientists include various (proxy) variables related to the animals’ biological functioning and their ability to cope with stress [10]. Available PHFWD studies focused on a selected and variable set of animal welfare parameters: production variables, mortality, physiological development, immunological variables and resistance to disease challenges. Some dimensions of welfare, especially behaviour and patho-physiological aspects (e.g., morbidity) have not been studied in great detail in relation to PHFWD. In addition, very little information is available concerning effects on behaviour or stress during the fasting period immediately post-hatch.

In order to determine whether or not PHFWD affects poultry welfare, it is important to consider both short-term (days) and long-term (weeks) effects, as there may be compensatory growth and/or altered physiological development following PHFWD. It should be noted, however, that there are fewer studies examining long-term effects than those examining relatively short-term effects of PHFWD. Additionally, it remains uncertain whether or not a delay in development has adverse consequences for bird welfare, e.g., through an increase in disease susceptibility.

### Production variables and mortality

Delayed access to food and water after hatching causes weight loss during holding (deprivation period), mainly due to dehydration and utilisation of yolk, while body weight starts to increase as soon as the chickens receive food and water [1, 4, 78, 79]. The MA showed that not only does PHFWD have short term effects on production and mortality, but that these effects were also found much later, even at six weeks (broilers’ slaughter age). Body weight and food intake decreased with increasing PHFWD. Initially, during the first week, FCR and mortality were higher when PHFWD was ≥84 hours, whereas total mortality at day 42 was already higher after 48 hours PHFWD compared to early-fed chickens. The effects of PHFWD on production variables and mortality may differ between studies applying treatments post-hatch (category 1) or post-pulling (category 2). Therefore, category 1 and 2 papers were analysed separately and together with category 3. In Category 2 papers the early- and mid-term hatching chickens are subjected to considerably longer periods of PHFWD than the late hatchers. This is not the case with category 1 papers where all chickens were fed at the same age after hatching. This also applies to the control group (0 hours PHFWD after hatching in category 1 and after pulling in category 2). However, separate analyses of production variables for Category 1 and 2 generally led to similar conclusions, despite the fact that the effect of PHFWD was larger in category 2 papers (e.g., reporting a numerically lower body weight after PHFWD than in category 1 papers) (data not shown). This suggests a negative effect on performance and mortality with a prolonged period of PHFWD, but there is no evidence for an exact ‘cut-off point’ after which negative effects start to occur, based on the existing literature.

The MA indicated that a very long duration of PHFWD (≥84 hours) had a pronounced negative effect on all production variables, including a large increase in total mortality compared to shorter durations. However, it is notable that this longer PHFWD was only applied in (old) category 3 studies, where the exact age of the chickens was unknown. Therefore, the exact duration of PHFWD remains uncertain; it is plausible that early hatching chickens were included and handling and transport occurred before the experiments started (e.g., [39]). It is acknowledged that the number of studies including a long PHFWD duration was small. Nevertheless, the results of the analysis indicate that a duration of ≥84 hours PHFWD is beyond the ‘cut-off point’ at which PHFWD adversely affects performance. Common hatchery procedures and transport may take periods up to and above 50 hours of PHFWD until arrival at the farm, and even up to 72 hours if longer transportion distances are involved [2, 3]. This would then suggest that a deprivation of ≥84 hours is probably rare in practice.

However, 48 hours PHFWD is likely to occur more frequently in commercial practice, and this could have long-term adverse effects on growth and mortality. The MA indicated that up to 42 days of age there is no full compensation in body weight gain and food intake although not all studies support this finding. It remains unclear whether or not the reduced growth and food intake following PHFWD is fully attributable to the delayed onset of growth, or is also affected by impaired subsequent development. The former is supported by extrapolating data of early-fed and delayed-fed chickens [80]. In addition to showing that 24 hours PHFWD (≥12–36 hours) (body weight) or 48 hours (≥36–60 hours) (food intake) have long-term effects on performance one of our main findings was that PHFWD of 48 hours has a long-term adverse effect on chicken mortality.

Only three papers reported effects of early access to water only (i.e. compared to deprivation of water) after hatching [20, 34, 77]. These studies indicated that broilers and turkey poults provided with water immediately after hatching had a higher body weight up to day 7 compared to water deprived and/or water and food deprived birds, but for water deprivation alone (without food deprivation) no long lasting effects were found on body weight [20, 34] and mortality [34]. Thus, the transient responses to water intake immediately after hatching are suggested to represent enhanced hydration without long term effects [7]. This is in contrast to the effects of food deprivation as reported in our MA. We did not find a single effect of food deprivation being dependent on the provision of water immediately after hatching, but, it is acknowledged that there are few studies reporting separately on the effects of water and food deprivation.

### Physiological variables

#### Relative yolk sac weight

At approximately day 18 of incubation, the remaining part of the yolk, which has not yet been utilised, is absorbed into the abdominal cavity. This residual yolk provides immediate nutrition for maintenance and growth after hatching [45, 81, 82]. During the first days post hatching, the chick makes the transition from utilizing energy in the form of yolk lipids to exogenous carbohydrate-rich food [65, 83]. It has been suggested that in the presence of food, the major route of yolk utilisation is via the yolk stalk into the small intestine, whereas in case of PHFWD, yolk is mainly resorbed directly into the blood via the yolk-sac membrane [84]. Thus, early post-hatch feeding may stimulate yolk sac resorption in the intestine (e.g., [7, 12, 48, 60]), although studies also found that early post-hatch feeding did not stimulate yolk sac resorption (e.g., [14, 16, 17, 40, 50]). Based on these ambiguous results, the effects of PHFWD on yolk sac resorption remain obscure.

The inconclusive results of yolk sac resorption were confirmed by the results of our MA, showing that 24 and 48 hours PHFWD did not significantly affect relative yolk sac weight at 3 days of age. The lower relative yolk sac weight after 72 hours PHFWD compared to early post-hatch feeding and drinking suggests that chickens subjected to long deprivation used the yolk sac for energy supply [39].

#### Relative organ weights

Relative organ weights are usually assessed in poultry as indicators of physiological development [85]. Several studies have reported effects of PHFWD on the relative weights of digestive organs of the chicken, such as the intestines (see next paragraph), liver, proventriculus, gizzard and pancreas (e.g., [12, 39, 59, 85]). It can be speculated that PHFWD retards the growth of digestive organs.

Few records indicated a significant negative effect (lower values) of PHFWD on relative liver weight during the first week of age. However, long-term effects (up to six weeks of age) were not observed in the majority of studies. Comparable results were found for relative pancreas and heart weights.

Relative gizzard and proventriculus weights were not included in the QA, because insufficient records were available. Cengiz et al. [62] showed lower relative gizzard weight at 10 days of age for 36 hours PHFWD compared to immediately fed chickens. Chickens fed immediately after hatching had higher relative stomach weights at pulling than PHFWD chickens [67]. Maiorka et al. [59] found that food deprived chickens that had received ad libitum water post-hatch, displayed lower relative proventriculus and gizzard weights after 48 hours and 72 hours of fasting compared to fed chickens. This was not observed in chickens fasted for 24 hours. Additionally, 56 hours PHFWD poults displayed lower gizzard weights at day 7 compared to non-deprived poults [37].

These results suggest only limited long-term effects of post-hatch food deprivation on the relative weights of heart, gizzard, proventriculus, liver and pancreas. Although during the fasting period and the first week of life relative organ weights were lower in several studies, the transient effect of PHFWD appears to be due to a delay in food intake.

#### Intestinal development

In newly hatched chickens, the gastrointestinal tract is immature, but develops physically (e.g., weight and length), morphologically (villus height and area, maturation of enterocytes and goblet cells, organisation and establishment of the crypt region) [83, 86], and physiologically (e.g., enzym activity and absorptive capacity) in the first weeks of age [83, 86, 87]. It has been suggested that yolk contributes to the small intestinal maintenance and development during the initial 48 hours post-hatch [65, 83, 86]. In addition, the intake of exogenous food results in a more rapid development of the gastro-intestinal tract. Therefore, post-hatch feeding is critical for the development of the intestines [83] and may affect the digestibility and absorption of nutrients later in life. In the first weeks after hatching, the gut-associated lymphoid tissue (GALT) is also developing, which suggests that PHFWD might affect the immunological development [83].

Many studies included measures of intestinal development to assess the effect of PHFWD (e.g., [14, 28, 37, 52, 59]). According to the QA, several records indicated a negative effect (numerically lower values) of PHFWD on duodenal, jejunal and ileal length, but no long-term effect was found. Villus height at week 1 or 2 of age was lower in some studies after PHFWD compared to immediately fed chickens, whereas lower crypt depths were mainly found for jejunum in the first weeks of life. However, some positive effects (higher values) of PHFWD on intestinal development were also observed and several studies failed to indicate any effect. It has been suggested that PHFWD can be more influential in the development of certain intestinal segments. Geyra et al. [28] and Uni et al. [33] observed delayed development in the duodenum and jejunum after PHFWD, but not in the ileum. The QA also points in this direction, with most significant effects found in the jejunum.

Besides the variables analysed in the QA, other variables related to intestinal development, such as crypt size, villus/crypt ratio, crypt proliferation, villus area and the rate of enterocyte migration [28], mucosal enzyme activity [33], goblet cell development [73], and mucosal aspects [62] have been investigated in relation to PHFWD. Unfortunately, insufficient records were available to include these in the QA.

It can be concluded that the effects of PHFWD on intestinal development appear to be transient. This does not mean that secondary effects (resulting from impaired digestion or absorption capabilities, e.g., impaired body weight gain) can not be long-lasting.

#### Hormones and plasma glucose

Results for plasma corticosterone levels in fasted chickens, immediately prior to the onset of post-hatch feeding, or immediately after feeding, are inconclusive [40, 67, 68, 88]. No long-term effects on plasma corticosterone concentration were found [40]. Plasma corticosterone concentrations are often measured as an indicator of stress and thus potential welfare problems. However, since corticosterone is also involved in the regulation of metabolism (blood glucose levels) and may respond to fasting (e.g., [85]), caution is advised when indicating that elevated plasma corticosterone levels associated with post-hatch food deprivation may be indicative of reduced welfare, though fasting itself is regarded as a stressor.

QA showed that plasma T3 (tri-iodothyronine) levels were lower during the period of food deprivation compared to early fed chicks, and T3 increased after feeding to similar levels as in early-fed control chickens [1, 68, 89]. Lower T3 values indicate a lower metabolic rate in fasting birds [1]. Plasma T4 was measured in only one study where no effect of PHFWD was reported [68].

Plasma glucose has been measured as an indicator of energy homeostasis, sometimes together with e.g., lactate [67, 68], protein or triglyceride levels [16]. The QA suggested that after 24 hours PHFWD plasma glucose levels were lower during the first two weeks of life compared to 0 hours PHFWD [16, 67, 68]. This may indicate a physiological need for energy, but it is questionable whether or not this will remain so for two weeks. Alternatively, 24 hours of PHFWD may have triggered a glucose holding mechanism, reflected in a lowering of the plasma concentration, that is maintained for two weeks after feed provision.

#### Immunology related variables

Several studies examined effects of PHFWD on (the development of) the immune system and the chickens’ ability to respond to challenges [27, 32, 35, 41, 44, 61, 72, 90–93]. However, insufficient records were available to facilitate the performance of a MA or QA on the variety of immunological variables. Two studies on GALT development suggest potential susceptibility to environmental pathogens of chickens with delayed access to food [27, 61].

Few studies have examined the responses to vaccines or disease model challenges, such as a coccidiosis vaccine challenge [27], a non-infectious lung challenge [44], an IBDV vaccine [41], Newcastle Disease vaccination [92], and a *Clostridium perfringens* challenge [35]. These studies indicate that PHFWD appears to have adverse consequences for the immune response to infectious disease challenges later in life [35, 41, 44, 94].

#### Effects of PHFWD in relation to genetic selection

Under natural conditions where a clutch of eggs is hatching under a broody hen, where the first chicks are hatching much earlier than the last ones, and where there is a strong biological (i.e. survival) need to leave the nest simultaneously, the yolk stores in the newly-hatched chick should be sufficient for energy and water supply to survive for approximately three days without ingesting food or water (in the absence of excessive thermoregulatory demands). However, it has been suggested that because of the high metabolic rate in fast growing broiler strains yolk sac reserves are depleted much more rapidly [6]. Indeed, fast growing broiler strains have a higher embryonic metabolic rate compared to slower growing strains [95], and it has also been shown that yolk sac resorption was faster in fast growing lines compared to slower growing lines or layer strains [17, 96, 97]. In addition, it has been suggested that food intake behaviour develops more rapidly in broiler chickens than jungle fowl [97]. Nielsen et al. [71] showed that post-hatch eating behaviour was delayed for approximately 4 hours in broilers from a slower growing strain compared to a fast growing strain. Thus the yolk sac reserves may be depleted more rapidly in modern fast growing broiler strains after fasting than in slower growing strains, but this merits further study.

Few studies have examined the relationship between PHFWD and genetic strain. Gonzales et al. [17] found that 36 hours PHFWD compared to 8 hours had negative effects on body weight at day 7 for fast growing broiler chickens, but not for laying hen chickens or a slower growing broiler strain. However, Simon et al. [32] subjected broilers and layers to 72 hours PHFWD and found a lower body weight gain up to 35 days of age, but no difference between breeds. Zhao et al. [26] did find differences between strains, but the implications are not clear, e.g., blood glucose leves increased and relative pancreas weights reduced following 72 hours PHFWD in slow growing broiler chickens compared to fast growing chickens.

#### Timing of voluntary post-hatch food and water intake

Under natural conditions, when chickens are raised by a mother hen, the first food eaten by the chick will be offered to it by the mother hen [98]. In the absence of a mother hen, newly hatched chickens peck indiscriminately at non-food and food objects, starting as early as a few hours after hatching [99, 100]. At three days of age pecks are directed primarily at the food [100]. From hatching until day 3, the pecking behaviour of newly-hatched chickens seems to be independent of the nutritional state [101]. By pecking at edible and non-edible objects, chickens learn to discriminate between food and non-food objects [102]. If so, this would seem to imply that food intake is rewarding to newly-hatched chickens, thus suggesting that early food intake may improve bird welfare despite the fact that perhaps PHFWD may itself not be directly having a negative effect on welfare (i.e. pecking behaviour being independent of nutritional state).

The mother hen does not appear to have a role in attracting the chicks to water. Newly-hatched chicks show an innate response to peck at shining objects and appear to seek out where to find water. They also recognise drinking behaviour of other chickens and are attracted to it [99, 103]. These studies, performed in non-commercial breeds or jungle fowl, again seem to suggest that the early provision of water would enhance chick welfare.

To our knowledge there are limited publications on the timing of voluntary first food and water intake of newly-hatched commercial chickens. Nielsen et al. [71, 104] observed broiler chickens showing pecking directed towards food particles on day 1 post-hatch, but the moment of actual first food intake was not mentioned. Pinchasov et al. [22] found that broiler chicks with immediate access to food and water consumed 1.5 g of food during the first 24 h. Although it is likely that the first food intake starts very early, probably within a couple of hours after hatching, the exact timing of (and factors affecting this) first food and water intake of commercial broiler breeds when water and food are available early post-hatch remains unknown.

It remains unclear whether or not PHFWD actually reduces welfare, due to the unfulfilled behavioural and physiological need for food intake post-hatch. A single study showed more chicks jumping and displaying active wakefulness and fewer chicks displayed sitting behaviour as duration of PHFWD increased. This was interpreted as an increased searching motivation for food and water in chickens subjected to a longer period of food deprivation [105]. This finding may indicate reduced welfare (stress or frustration) despite the earlier suggestion (no adverse welfare impact) related to the finding that pecking is independent of nutritional state [101]. This requires further investigation.

#### Variation in effects of PHFWD

As demonstrated in both the MA and QA, considerable variation was found among studies in the measurable effects of PHFWD. This may therefore suggest that other factors may be interfering. S1 Table provides a list of these factors and a brief discussion how they can influence effecs of PHFWD.

Furthermore, due to a large variation in the duration of PHFWD among studies, classes of deprivation times were used and compared to the 0 hours deprivation treatment (or early-fed chickens). This implies that reported effects cannot be translated directly into a ‘cut-off point’, because of the range of PHFWD within each class. For example, 72 hours PHFWD class includes the time span from ≥60 to 84 hours, but it remains unclear whether or not the ‘cut-off point’ for effects of PHFWD on performance, development and welfare is 60 or 84 hours, or in between. In addition, studies differed with respect to the degree to which they reported the real age of the chickens, i.e. biological age, chronological age, and ‘age unclear’ (categories 1–3, respectively). In the case of the use of chronological age, this could imply that chickens are even longer deprived of food and water after hatching than was indicated in the study, because chickens hatch within a window of 24 to 36 hours [1] and those chickens that hatched first are deprived longer of food and water than those hatching later.

## Conclusions

The MA showed that PHFWD (post-hatch food deprivation or food and water deprivation) for 24 hours (i.e. ≥12–36 hours) resulted in significantly lower body weights compared to early fed chickens up to six weeks of age. Body weights and food intake were reduced more as PHFWD durations (24, 48, 72, ≥84 hours) increased. However, it is unclear whether or not effects of PHFWD on body weight are due to impaired development or to a delayed onset of growth. FCR at 21 and 42 days of age was increased from 48 hours PHFWD onwards. Although single studies did not find effects of PHFWD on mortality, the MA showed that 48 hours PHFWD leads to significantly higher total mortality at six weeks of age than 0 or 24 hours PHFWD.

The QA results indicate a transient negative effect of PHFWD on the development of liver and pancreas, and that PHFWD may delay the development of duodenum, jejunum and ileum. These effects were observed mainly in the first week of age. Short term (potentially adverse) changes in plasma T3 and glucose concentrations were also found.

With respect to the effect of PHFWD on poultry welfare, the higher total mortality at 48 hours PHFWD suggests compromised welfare, as does the delayed growth and multiple other findings discussed in this paper. However, for a final conclusion with respect to welfare, additional studies are recommended on the effect of PHFWD containing a wider range of variables, including behaviour and disease resistance, in the short-term as well as long-term.

## Supporting information

S1 Fig QA results of relative organ weights. (PDF)

S2 Fig QA results of intestinal length. (PDF)

S3 Fig QA results of intestinal weight. (PDF)

S4 Fig QA results of villus height. (PDF)

S5 Fig QA results of crypt depth. (PDF)

S6 Fig QA results of plasma T3, T4 and glucose concentration. (PDF)

S1 Table Overview of biotic and abiotic factors that may interfere with the effects of post-hatch food water deprivation (PHFWD) on chick performance, development and welfare. (DOCX)

S2 Table Prisma checklist. (DOC)

S1 Data File Datafile part 1, gut aspects. (XLSX)

S2 Data File Datafile part 2, all variables except gut aspects. (XLSX)
