# Supplementary material for: A 'meta-analysis' of effects of post-hatch food and water deprivation on development, performance and welfare of chickens
Source: PLoS One. 2017 Dec 13;12(12):e0189350. doi: 10.1371/journal.pone.0189350 (PMC5728577; doi:10.1371/journal.pone.0189350)
Supplement: S3 Fig — (PDF) [file pone.0189350.s003.pdf]

S3 Fig. Qualitative analysis results of intestinal weight.

Number of records demonstrating positive (numerically higher values), negative (numerically lower values) or no effects (NS) of post-hatch food and water deprivation for 24, 48 or 72 hours compared to 0 hours deprivation on relative duodenal (A), jejunal (C) and ileal (E) weight from day 1-6 of age or relative duodenal (B), jejunal (D) and ileal (F) weight between 1-6 weeks of age.

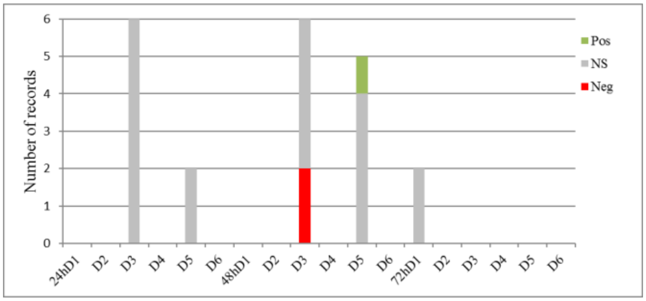

A. Duodenal weight

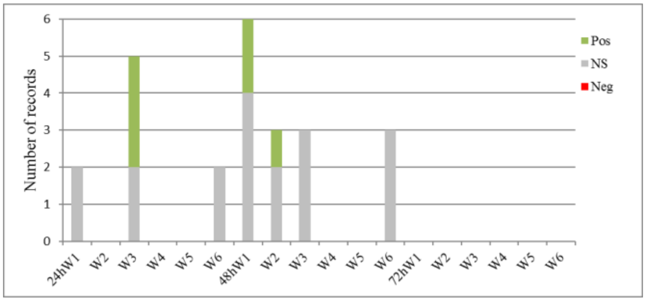

B. Duodenal weight

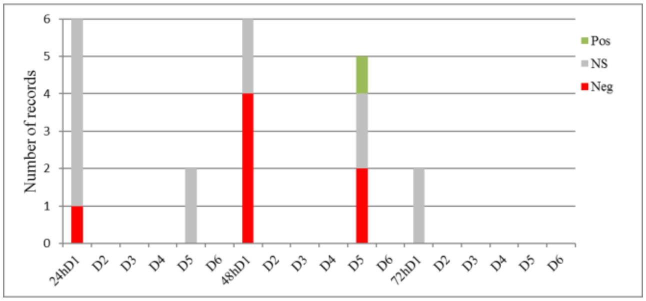

C. Jejunal weight

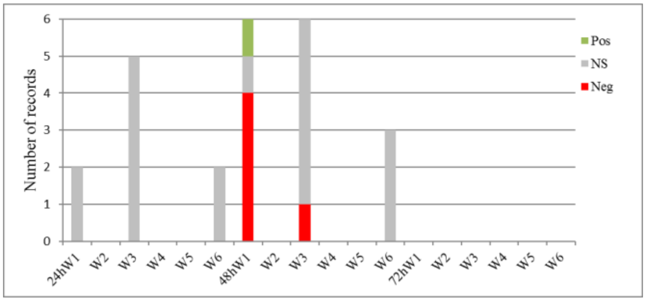

D. Jejunal weight

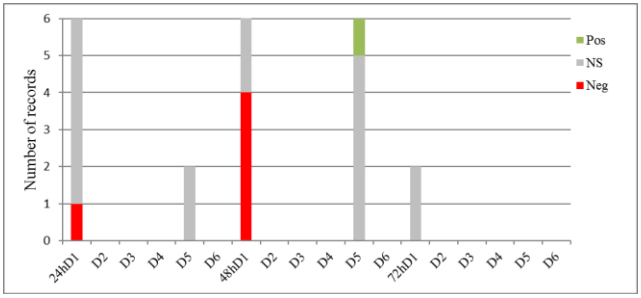

E. Ileal weight

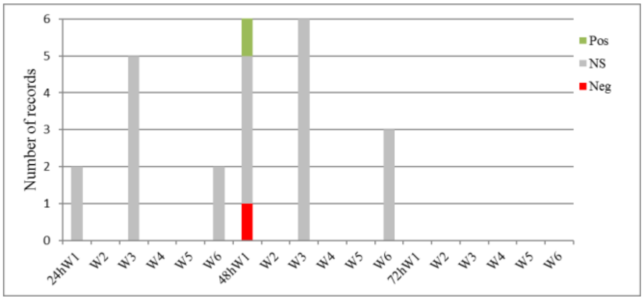

F. Ileal weight
