# Supplementary material for: A 'meta-analysis' of effects of post-hatch food and water deprivation on development, performance and welfare of chickens
Source: PLoS One. 2017 Dec 13;12(12):e0189350. doi: 10.1371/journal.pone.0189350 (PMC5728577; doi:10.1371/journal.pone.0189350)
Supplement: S6 Fig — (PDF) [file pone.0189350.s006.pdf]

S6 Fig. Qualitative analysis results of plasma T3, T4 and glucose concentration.
Number of records demonstrating positive (numerically higher values), negative (numerically lower values) or no effects (NS) of post-hatch food and water deprivation for 24, 48 or 72 hours compared to 0 hours deprivation on plasma T3 concentration between 1-6 days of age (A), and plasma glucose concentration between 1-6 days of age (B) or 1-6 weeks of age (C).

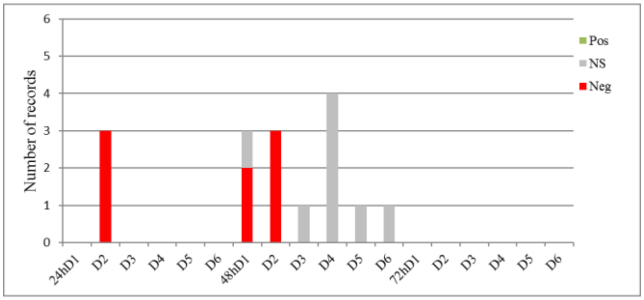

A. T3

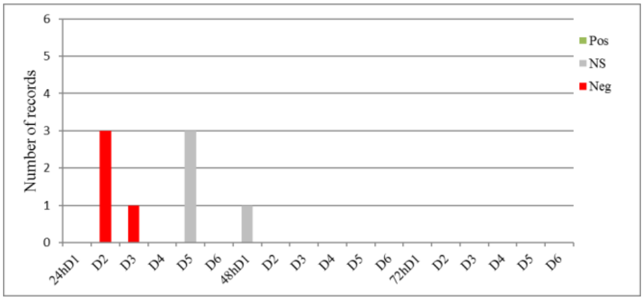

B. Glucose

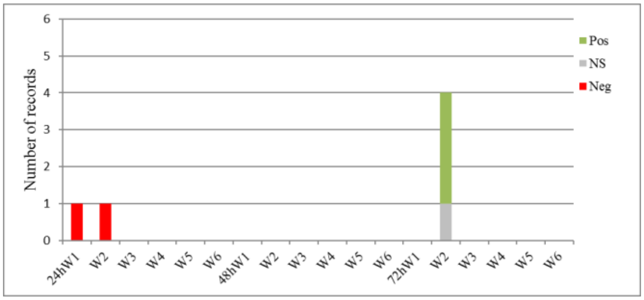

C. Glucose
