# Supplementary material for: A 'meta-analysis' of effects of post-hatch food and water deprivation on development, performance and welfare of chickens
Source: PLoS One. 2017 Dec 13;12(12):e0189350. doi: 10.1371/journal.pone.0189350 (PMC5728577; doi:10.1371/journal.pone.0189350)
Supplement: S1 Table — (DOCX) [file pone.0189350.s007.docx]

**S1 Table.**

**Table. Overview of biotic and abiotic factors that may interfere with effects of post-hatch food and water deprivation on chicken performance, development and welfare^1^.**

| Biotic factors^2^ | Abiotic factors^3^ |
| --- | --- |
| - Type (e.g. layers vs. broilers) | - Storage duration of eggs before incubation |
| - Cross/line (different genetic lines within layers or broilers) | - Incubation temperature |
| - Age of the parent stock | - Incubation relative humidity |
| - Egg size | - Incubation gas concentrations |
| - Hatching time | - Diet composition in early life - Transport (duration, quality)* - Vaccination protocol* - Housing temperature and ventilation* - Other environmental conditions (noise, vibrations, dust)* - Disturbances (e.g. inspections by people)* - Food (and perhaps water) quality, physical presentation, presence (accessibility; way of presentation)* |
| - Sex |  |

^1^For several factors mentioned in the table above (marked *), such as vaccination protocol, transport [1] and housing temperature [2], relatively little if anything is known in relation to post-hatch food and water deprivation. Therefore, these factors will not be discussed below.

^2^ Biotic factors, brief explanatory notes:

- Type: layers and broilers are selected for different production purposes, which has led to physiological differences during incubation and post-hatching. For example, differences exist in egg composition [3], resulting in differences in chick weight, residual yolk weight, and organ development (e.g. [3-6]). Whether these differences in embryonic development are associated with differences in chicken quality has not been established yet. Yet, available scientific knowledge indicates that broiler and layer chickens possibly differ in their response to early or delayed feeding (see also the previous section).
- Strain/cross: comparisons between layer lines on chick quality have hardly been made, but comparisons between broiler lines have been investigated more often. Nangsuay et al. [7, 8] concluded that Ross 308 embryos had a lower nutrient efficiency, produce more heat during late incubation, and hatch later than Cobb500. In addition, Cobb500 broilers were more vulnerable to overheating during incubation (see abiotic factors). Also O’Dea et al. [9], Hamidu et al. [10] and Tona et al. [11] demonstrated differences between broiler breeds in egg composition, embryonic development and hatching time. This might affect chicken quality and therefore it can be suggested that different breeds will respond differently to post-hatch food and water deprivation (see also the previous section).
- Age of the parent stock: older breeders in general produce larger eggs, but even when eggs of the same size of young and old breeders are compared, it has been shown that egg composition differs between eggs from young and old breeders [12-14]. However, this does not automatically lead to differences in chick weight at hatching, but differences in chick composition will probably occur. Chickens obtained from older breeders demonstrated to have more protein and fat deposition in the body [12], which might make them less vulnerable after hatching. For example, Weijntjes et al. [15] showed that chickens of older breeders are less sensitive to low ambient temperatures during the early post-hatch period than chickens of younger breeders. Breeder age may modulate the effects of early food deprivation [16, 17], and because of that differences between studies on post-hatch food and water deprivation may be related to differences in age of the breeder [18, 19].
- Egg size: The size of the egg, even within a given breeder age, largely determines the weight of the hatchling. Larger eggs produce more heat during incubation [20] and produce heavier hatchlings [12, 20], but also the protein and fat deposition in the body differs [12, 20]. This suggests that chickens of larger eggs are more mature at hatching and have other nutritional and environmental needs. Again, it is not clear whether egg size and consequently hatchling weight indeed influence effects of post-hatch food and water deprivation.
- Hatching time: Physiological differences exist between hatchlings of the same hatch. It has been shown that early, midterm and late hatching chickens differ physiologically [21-26]. This means that effects of delayed feeding may not only depend on the duration of post-hatch food deprivation, but also on the differences in hatching moment (early/midterm/late hatching) due to the differences in physiological status of the birds. As has been demonstrated, early, midterm and late hatching chickens might differ in their response to delayed feeding [22-24]. It is thus important to consider this aspect when comparing studies that provided food immediately after hatching compared to studies that provided food immediately after pulling.
- Sex: Male and female chickens hardly differ in their embryonic development, although female chickens in general hatch somewhat earlier than male chickens [27]. Because in some studies on delayed or early feeding only one sex of chickens was used, it might be that the applied treatments are partially related to that specific sex. However, the aspect of difference in hatching time between females and males is hardly taken into consideration in experiments.

^3^ Abiotic factors, brief explanatory notes:

- Storage duration of eggs before incubation: Eggs are sometimes stored for up to 14 days before incubation is started. Prolonged storage has been shown to negatively affect chicken quality at hatching and later life performance in terms of growth [28-30]. It can be speculated that differences could existed between studies in storage duration and that there are differences in chicken quality at hatching. Furthermore, it can be speculated that chickens obtained from prolonged stored eggs, differing in residual yolk weight at hatching, will respond differently to delayed or early feed provision than chickens obtained from eggs incubated shortly after oviposition. For example, Careghi et al. [31] showed that long duration of egg storage depressed relative growth, not only in chickens with immediate access to food, but also in those subjected to post-hatch food deprivation, but this growth depression was significantly aggravated in eggs subjected to long storage time.
- Incubation temperature: A very important factor affecting chicken quality at hatching is the incubation temperature. For example, Lourens et al. [32], Molenaar et al. [33], and Maatjens et al. [34, 35] demonstrated strong effects of incubation temperature on chicken quality at hatching. Furthermore, e.g. Hulet et al. [36], Leksrisompong et al. [37] and Molenaar et al. [33] showed that incubation temperature may have long-term effects on growth and development in later life. Particularly, a high incubation temperature during the last week of incubation, that quite often occurs in practice [38] appears to affect chicken quality, particularly when the period between hatching and pulling is prolonged, due to increased body weight loss as a result of evaporation. However, the interaction between incubation temperature and post-hatch food and water deprivation has to our knowledge not been investigated yet.
- Relative humidity: Relative humidity in the incubator is affecting egg weight loss and consequently chick quality. In general, it is assumed that egg weight loss between the start of incubation and day 18 of incubation should be between 12 and 14% for optimal results. Van der Pol et al. [2] demonstrated that relative humidity is of less importance when the incubation temperature remains optimal. However, particularly in relation to chick quality, the period between hatching and pulling is of interest. When the relative humidity in the incubator is low, chickens will lose more water via evaporation and consequently chick quality may be affected. Possibly, chickens that are more dehydrated before pulling, will be able to cope less well with post-hatch food and water deprivation than non-dehydrated chickens.
- Incubation gas concentrations: During incubation, embryos use oxygen to metabolise egg nutrients and to convert them into body tissues. Consequently, carbon dioxide is produced. Both gasses may affect chick quality. Lourens et al. [39] and Molenaar et al. [40] demonstrated that lower oxygen concentrations than normal resulted in lower yolk free body weight and higher residual yolk weight at hatching. This phenomenon might also play a role when experiments are carried out at high altitude. Additionally, high carbon dioxide concentrations during late incubation may affect hatching time (e.g. [41]), which may affect chick quality at hatching. It can be speculated that both oxygen concentration and carbon dioxide concentration during incubation can interact with delayed or early post-hatch feeding, but this has not been demonstrated in scientific literature yet.
- Diet composition: A factor that does not affect chicken quality at hatching itself, but might explain differences among studies on post-hatch food and water deprivation is the diet composition [42]. Effects of diet composition in combination with delayed or early food provision has hardly been examined, but based on the limited studies done in this field [43, 44], it appears that diet composition may indeed play a role. In studies on post-hatch food and water deprivation, food composition is often not specified. However, it probably varies considerably among studies, which could explain differences in the outcomes among studies on post-hatch food and water deprivation.

**References**

1. Bergoug H, Guinebretière M, Tong Q, Roulston N, Romanini C, Exadaktylos V, et al. Effect of transportation duration of 1-day-old chicks on postplacement production performances and pododermatitis of broilers up to slaughter age. Poultry science. 2013;92(12):3300-9. doi: 10.3382/ps.2013-03118.

2. van der Pol CW, van Roovert-Reijrink IAM, Maatjens CM, van den Brand H, Molenaar R. Effect of relative humidity during incubation at a set eggshell temperature and brooding temperature posthatch on embryonic mortality and chick quality. Poultry Science. 2013;92(8):2145-55. doi: 10.3382/ps.2013-03006.

3. Nangsuay A, Molenaar R, Meijerhof R, van den Anker I, Heetkamp MJW, Kemp B, et al. Differences in egg nutrient availability, development, and nutrient metabolism of broiler and layer embryos. Poultry Science. 2015;94(3):415-23. doi: 10.3382/ps/pev007.

4. Everaert N, Willemsen H, De Smit L, Witters A, De Baerdemaeker J, Decuypere E, et al. Comparison of a modern broiler and layer strain during embryonic development and the hatching process. British Poultry Science. 2008;49(5):574-82.

5. Janke O, Tzschentke B, Boerjan M. Comparative investigations of heat production and body temperature in embryos of modern chicken breeds. Avian and Poultry Biology Reviews. 2004;15(3-4):191-6. doi: 10.3184/147020604783637868.

6. Sato M, Tachibana T, Furuse M. Heat production and lipid metabolism in broiler and layer chickens during embryonic development. Comparative Biochemistry and Physiology a-Molecular & Integrative Physiology. 2006;143(3):382-8. doi: 10.1016/j.cbpa.2005.12.019.

7. Nangsuay A, Meijerhof R, van den Anker I, Heetkamp MJW, Kemp B, van den Brand H. Development and nutrient metabolism of embryos from two modern broiler strains. Poultry Science. 2015;94(10):2546-54. doi: 10.3382/ps/pev234.

8. Nangsuay A, R. Meijerhof R, van den Anker I, Heetkamp MWJ, Kemp B, Van den Brand H. Effects of breeder age, strain, and eggshell temperature on nutrient metabolism of broiler embryos. Poultry Science. 2017;96(6)1891-1900.

9. O'Dea EE, Fasenko GM, Feddes JJR, Robinson FE, Segura JC, Ouellette CA, et al. Investigating the eggshell conductance and embryonic metabolism of modern and unselected domestic avian genetic strains at two flock ages. Poultry Science. 2004;83(12):2059-70.

10. Hamidu J, Fasenko G, Feddes J, O’dea E, Ouellette C, Wineland M, et al. The effect of broiler breeder genetic strain and parent flock age on eggshell conductance and embryonic metabolism. Poultry Science. 2007;86(11):2420-32. doi: 10.3382/ps.2007-00265.

11. Tona K, Onagbesan OM, Kamers B, Everaert N, Bruggeman V, Decuypere E. Comparison of Cobb and Ross strains in embryo physiology and chick juvenile growth. Poultry Science. 2010;89(8):1677-83. doi: 10.3382/ps.2009-00386.

12. Nangsuay A, Meijerhof R, Ruangpanit Y, Kemp B, van den Brand H. Energy utilization and heat production of embryos from eggs originating from young and old broiler breeder flocks. Poultry Science. 2013;92(2):474-82. doi: 10.3382/ps.2012-02643.

13. Nangsuay A, Meijerhof R, Van den Anker I, Heetkamp MJW, Morita VD, Kemp B, et al. Effects of breeder age, broiler strain, and eggshell temperature on development and physiological status of embryos and hatchlings. Poultry Science. 2016;95(7):1666-79. doi: 10.3382/ps/pew080.

14. Nangsuay A, Ruangpanit Y, Meijerhof R, Attamangkune S. Yolk absorption and embryo development of small and large eggs originating from young and old breeder hens. Poultry Science. 2011;90(11):2648-55. doi: 10.3382/ps.2011-01415.

15. Weytjens S, Meijerhof R, Buyse J, Decuypere E. Thermoregulation in chicks originating from breeder flocks of two different ages. The Journal of Applied Poultry Research. 1999;8(2):139-45. doi: 10.1093/japr/8.2.139

16. Noy Y, Pinchasov Y. Effect of a single posthatch intubation of nutrients on subsequent early performance of broiler chicks and turkey poults. Poultry Science. 1993;72(10):1861-6.

17. Vargas FSC, Baratto TR, Magalhaes FR, Maiorka A, Santin E. Influences of breeder age and fasting after hatching on the performance of broilers. Journal of Applied Poultry Research. 2009;18(1):8-14. doi: 10.3382/japr.2008-00029.

18. El Sabry MI, Yalcin S, Turgay-Izzetoglu G. Interaction between breeder age and hatching time affects intestine development and broiler performance. Livestock Science. 2013;157(2-3):612-7. doi: 10.1016/j.livsci.2013.07.012.

19. Mahmoud KZ, Edens FW. Breeder age affects small intestine development of broiler chicks with immediate or delayed access to feed. British Poultry Science. 2012;53(1):32-41. doi: 10.1080/00071668.2011.652596.

20. Lourens A, Molenaar R, van den Brand H, Heetkamp MJW, Meijerhof R, Kemp B. Effect of egg size on heat production and the transition of energy from egg to hatchling. Poultry Science. 2006;85(4):770-6.

21. Lamot DM, van de Linde IB, Molenaar R, van der Pol CW, Wijtten PJA, Kemp B, et al. Effects of moment of hatch and feed access on chicken development. Poultry Science. 2014;93(10):2604-14. doi: 10.3382/ps.2014-04123.

22. van de Ven LJF, van Wagenberg AV, Debonne M, Decuypere E, Kemp B, van den Brand H. Hatching system and time effects on broiler physiology and posthatch growth. Poultry Science. 2011;90(6):1267-75. doi: 10.3382/ps.2010-00876.

23. van de Ven LJF, van Wagenberg AV, Decuypere E, Kemp B, van den Brand H. Perinatal broiler physiology between hatching and chick collection in 2 hatching systems. Poultry Science. 2013;92(4):1050-61. doi: 10.3382/ps.2012-02534.

24. Wang Y, Li Y, Willems E, Willemsen H, Franssens L, Koppenol A, et al. Spread of hatch and delayed feed access affect post hatch performance of female broiler chicks up to day 5. Animal. 2014;8(4):610-7. doi: 10.1017/s175173111400007x.

25. Powell DJ, Velleman SG, Cowieson AJ, Singh M, Muir WI. Influence of chick hatch time and access to feed on broiler muscle development. Poultry Science. 2016;95(6):1433-48. doi: 10.3382/ps/pew047.

26. Powell DJ, Velleman SG, Cowieson AJ, Singh M, Muir WI. Influence of hatch time and access to feed on intramuscular adipose tissue deposition in broilers. Poultry Science. 2016;95(6):1449-56. doi: 10.3382/ps/pew063.

27. Van de Ven L, Van Wagenberg A, Debonne M, Decuypere E, Kemp B, Van Den Brand H. Hatching system and time effects on broiler physiology and posthatch growth. Poultry Science. 2011;90(6):1267-75. doi: 10.3382/ps.2010-00876.

28. Reijrink IAM, Berghmans D, Meijerhof R, Kemp B, van den Brand H. Influence of egg storage time and preincubation warming profile on embryonic development, hatchability, and chick quality. Poultry Science. 2010;89(6):1225-38. doi: 10.3382/ps.2009-00182.

29. Tona K, Bamelis F, De Ketelaere B, Bruggeman V, Moraes V, Buyse J, et al. Effects of egg storage time on spread of hatch, chick quality, and chick juvenile growth. Poultry Science. 2003;82(5):736-41. doi: 10.1093/ps/82.5.736.

30. Tona K, Onagbesan O, De Ketelaere B, Decuypere E, Bruggeman V. Effects of age of broiler breeders and egg storage on egg quality, hatchability, chick quality, chick weight, and chick posthatch growth to forty-two days. Journal of Applied Poultry Research. 2004;13(1):10-8.

31. Careghi C, Tona K, Onagbesan O, Buyse J, Decuypere E, Bruggeman V. The effects of the spread of hatch and interaction with delayed feed access after hatch on broiler performance until seven days of age. Poultry Science. 2005;84(8):1314-20.

32. Lourens A, Van den Brand H, Meijerhof R, Kemp B. Effect of eggshell temperature during incubation on embryo development, hatchability, and posthatch development. Poultry science. 2005;84(6):914-20. doi: 10.1093/ps/84.6.914.

33. Molenaar R, Hulet R, Meijerhof R, Maatjens CM, Kemp B, van den Brand H. High eggshell temperatures during incubation decrease growth performance and increase the incidence of ascites in broiler chickens. Poultry Science. 2011;90(3):624-32. doi: 10.3382/ps.2010-00970.

34. Maatjens C, Reijrink I, van den Anker I, Molenaar R, van der Pol C, Kemp B, et al. Temperature and CO2 during the hatching phase. II. Effects on chicken embryo physiology. Poultry science. 2014;93(3):655-63. doi: 10.3382/ps.2013-03491.

35. Maatjens C, van Roovert-Reijrink I, Engel B, van der Pol C, Kemp B, van den Brand H. Temperature during the last week of incubation. I. Effects on hatching pattern and broiler chicken embryonic organ development. Poultry science. 2016;95(4):956-65. doi: 10.3382/ps/pev447.

36. Hulet R, Gladys G, Hill D, Meijerhof R, El-Shiekh T. Influence of egg shell embryonic incubation temperature and broiler breeder flock age on posthatch growth performance and carcass characteristics. Poultry Science. 2007;86(2):408-12.

37. Leksrisompong N, Romero-Sanchez H, Plumstead PW, Brannan KE, Yahav S, Brake J. Broiler incubation. 2. Interaction of incubation and brooding temperatures on broiler chick feed consumption and growth. Poultry Science. 2009;88(6):1321-9. doi: 10.3382/ps.2008-00412.

38. Lourens A, Meijerhof R, Kemp B, Van den Brand H. Energy partitioning during incubation and consequences for embryo temperature: A theoretical approach. Poultry science. 2011;90(2):516-23. doi: 10.3382/ps.2010-00928

39. Lourens A, Van den Brand H, Heetkamp M, Meijerhof R, Kemp B. Effects of eggshell temperature and oxygen concentration on embryo growth and metabolism during incubation. Poultry science. 2007;86(10):2194-9. doi: 10.1093/ps/86.10.2194.

40. Molenaar R, Meijerhof R, van den Anker I, Heetkamp MJW, van den Borne J, Kemp B, et al. Effect of eggshell temperature and oxygen concentration on survival rate and nutrient utilization in chicken embryos. Poultry Science. 2010;89(9):2010-21. doi: 10.3382/ps.2010-00787.

41. Tong Q, McGonnell IM, Roulston N, Bergoug H, Romanini CEB, Garain P, et al. Higher levels of CO2 during late incubation alter the hatch time of chicken embryos. British Poultry Science. 2015;56(4):503-9. doi: 10.1080/00071668.2015.1041097.

42. Willemsen H, Debonne M, Swennen Q, Everaert N, Careghi C, Han H, et al. Delay in feed access and spread of hatch: importance of early nutrition. World's Poultry Science Journal. 2010;66(02):177-88. doi: <http://dx.doi.org/10.1017/S0043933910000243>.

43. Lamot DM, van der Klein SAS, de Linde IBV, Wijtten PJA, Kemp B, van den Brand H, et al. Effects of feed access after hatch and inclusion of fish oil and medium chain fatty acids in a pre-starter diet on broiler chicken growth performance and humoral immunity. Animal. 2016;10(9):1409-16. doi: 10.1017/s1751731116000288.

44. Van den Brand H, Molenaar R, Van der Star I, Meijerhof R. Early feeding affects resistance against cold exposure in young broiler chickens. Poultry science. 2010;89(4):716-20. doi: 10.3382/ps.2009-00432.
